# Current data on extremities chronic osteomyelitis in southwest China: epidemiology, microbiology and therapeutic consequences

**DOI:** 10.1038/s41598-017-16337-x

**Published:** 2017-11-24

**Authors:** Xiaohua Wang, Shengpeng Yu, Dong Sun, Jingshu Fu, Shulin Wang, Ke Huang, Zhao Xie

**Affiliations:** National & Regional United Engineering Laboratory of Tissue Engineering, Department of Orthopaedics, Southwest Hospital, Third Military Medical University, Chongqing, 400038 The People’s Republic of China

## Abstract

The current study was designed to explore the epidemiology of extremities chronic osteomyelitis, its prognosis and the complications of the treatment methods being used in southwest China. The data from osteomyelitis patients treated at the Department of Orthopaedics, Southwest Hospital, China between May 2011 and September 2016 were collected and analysed. The study comprised 503 admitted patients, of which 416 males and 87 were females, with an average age of 40.15 ± 5.64 years. Approximately 356 cases were followed for more than 18 months; the average bone union time was 6.24 ± 0.76 months in 94.1% (335) patients, and infections were almost controlled in 93.8% patients. The rate of infection control with the induced membrane technique was higher than with the I-stage free bone graft. Iliac infection was the main complication of the induced membrane technique, and impaired joint activity was the main complication of I-stage free bone grafts. In southwest China, the incidence of haematogenous osteomyelitis, caused mainly by *Staphylococcus aureus*, remains very high. The speed of bone defect repair and the rate of infection control with the induced membrane technique were superior to those of I-stage free bone grafts. Internal fixation should be given priority because it offers reduced complications with no increase in the recurrence of infection.

## Introduction

Osteomyelitis is a severe bone infection that results from various aetiologies and mechanisms^1^. With recent advancements in surgical techniques, the incidence of haematogenous osteomyelitis has decreased markedly, while the incidence of post-traumatic osteomyelitis has remained at higher levels. The incidence of osteomyelitis in the American population during the last decade (2000–2009) was twice that of 40 years ago^2^. Recent changes in the epidemiology, pathogenesis, diagnosis, treatment, and prognosis of this disease have varied according to population^3,4^. Clinical signs of osteomyelitis are diverse and are probably altered by geography, lifestyle and the quality of available medical services^5–7^. The phalanges bones are frequently affected^8^, particularly in African populations, but in many other countries, the most susceptible bones are the tibia or femur^1,9,10^. During a survey spanning 2005 to 2012, the incidence of acute haematogenous osteomyelitis (AHO) in southern Israel was 5.6:100,000, but in the Bedouin and Jewish populations, the incidences were 7.3 and 4.1:100,000, respectively^10^. Chronic bone infections are more often linked with diverse bacterial biofilms^11^.

China has a rich diversity of populations, climates, and lifestyles. Southwest China is underdeveloped in terms of economic growth compared with coastal China, and its very cloudy and humid climate offers a desirable niche for diverse microbial growth. In terms of osteomyelitis, southwest China is characterised by several features: i) the incidence of osteomyelitis is considerably higher than in other parts of China, ii) there is a shortage of well-trained physicians specialized in addressing bone infections, and iii) patients are forced to seek treatment in a few select hospitals. There are few prominent hospitals, which lack relevant reports and authentic data. In this context, we aimed to explore the clinical characteristics of osteomyelitis in southwest China and to observe the treatment and prognosis of this disease.

## Methods

A retrospective analysis of 503 osteomyelitis patients admitted to the Department of Orthopaedics, Southwest Hospital, China, from May 2011 to September 2016 was performed. We followed patients who met the diagnosis of osteomyelitis through the electronic medical records system to collect their basic information, history, clinical manifestations, auxiliary examinations, treatment, prognosis and complications.

### Inclusion criteria

patients diagnosed with osteomyelitis based on the following criteria: local bone pain and swelling on examination, draining fistula, imaging procedures, microbiological and histopathological examinations, and biochemical examinations.

### Exclusion criteria

patients with acute osteomyelitis (less than 2 weeks); patients without surgical treatment; osteomyelitis in the spine, pelvis and skull; patients with incomplete data; and patients with diabetic foot infection who were not treated in our department. Finally, 503 patients were selected. Of these, 320 were treated with the induced membrane two-stage technique, and 183 were treated with I-stage free bone grafts (bone grafts after debridement); no other methods were used in this study. Intravenous antibiotic therapy was applied for approximately 2 weeks post operation, third-generation cephalosporin (ceftazidime or cefpiramide) was administered to patients with a negative culture, and no patient received oral antibiotic treatment. Standard follow-up was performed every three months to observe infection control, bone healing and complications. The Ethics Committee of Southwest Hospital, Third Military Medical University Chongqing, China, approved this study, and any related procedures were performed in accordance with relevant guidelines and regulations.

### Ethics approval and informed consent

Ethics approval was obtained from the Southwest Hospital, Third Military Medical University Ethics Committee. All the participants gave their written informed consent.

## Statistics

Numerative data (sites, sinus, complications) were compared using Pearson’s chi-square test or Fisher’s exact test, while measurement data (age, levels of inflammation markers, bone union time) were compared using the t test or rank-sum test. Relevant risk factors were analysed using logistic regression analysis; to compare the influence of fixators on prognosis, we used one-way ANOVA or the chi-square test. P values below 0.05 were considered significant.

## Results

There were 503 patients, including 416 males and 87 females, with an average age of 40.15 ± 5.64 years. The average number of previous operations was 2.33 ± 0.22; 2.60 ± 0.27 times for those with post-traumatic osteomyelitis and1.14 ± 0.20 times for those with haematogenous osteomyelitis. The average duration of infection was 62.91 ± 11.38 months. The 503 patients experienced infections in 510 sites; the rate of sinus infection was 69.2% (353/510) and was higher among the post-traumatic osteomyelitis patients than among those with haematogenous osteomyelitis (P < 0.01). Forty-one patients had type 2 diabetes or other systemic adverse factors, 15 patients had varicose veins or other local adverse factors, and 169 patients had a history of smoking; the data are presented in Table 1. The use of contiguous seeding based on skin lesions, the number of cases was kept small enough to tabulate.

**Table 1:** Comparisons of different types of osteomyelitis.

Events	Post-Traumatic Osteomyelitis	Hematogenous Osteomyelitis	P Value
Number	403	90	—
Average age (years)	41.33 ± 5.65	35.41 ± 5.92	<0.01
Single site/multiply sites	398/5	89/1	>0.05
Most common single site	Tibia (242/398)	Femur (39/89)	<0.01
Most common Cierny-Mader type	IV 54.9% (224/408)	III 38.5% (35/91)	<0.01
Average duration of infection (months)	37.14 ± 9.05	174.93 ± 16.14	<0.01
Rate of sinus	74.8% (305/408)	49.5% (45/91)	<0.01
Rate of skin defects	24.8% (101/408)	7.7% (7/91)	<0.01
Averaged operation times	2.60 ± 0.27	1.14 ± 0.20	<0.01
Positive rate of culture	66.9% (273/408)	54.9% (50/91)	<0.01
Most common bacteria monomicrobial infection	Staphylococcus aureus 43.6% (102/234)	Staphylococcus aureus 76.2% (32/42)	<0.01
Rate of bone defect or nonunion	42.9% (175/408)	4.4% (4/91)	<0.01
**Serum levels of preoperative inflammation markers**			
WBC (thousands/microL)	7.49 ± 0.47	8.79 ± 0.39	<0.01
CRP (mg/L)	17.01 ± 2.33	40.14 ± 4.12	<0.05
ESR (mm/hr)	24.41 ± 1.88	36.5 ± 1.97	<0.01
**Positive rate of serum levels of preoperative inflammation markers**			
WBC	14.9% (60/403)	26.7% (24/90)	<0.01
CRP	41.1% (165/402)	54.4% (49/90)	<0.05
ESR	38.2% (153/401)	55.6% (50/90)	<0.01

WBC: White blood cell; CRP: C-reactive protein; ESR: Erythrocyte sedimentation rate.

### Infection site

Among the 496 cases with a single-site infection, there were 285 cases of tibia infection, 133 femur infections, 13 ulna and radius infections, 14 humerus infections, 11 fibula infections, 15 calcaneus infections, 7 clavicle infections, 6 metatarsal infections, 5 infections of the toes, 4 patella infections, and 3 phalanx infections. Seven patients had multiple ipsilateral infection sites. A total of 408 sites were infected in the post-traumatic osteomyelitis patients compared with 91 sites in the haematogenous osteomyelitis group. The right side accounted for 47.1% of the total infection sites, and the left side accounted for 52.9%.

### Classification

According to the Waldvogel classification, there were 403 cases (80.1%) of post-traumatic osteomyelitis, 90 cases (17.9%) of haematogenous osteomyelitis and 10 (2%) contiguous seedings from skin lesion infections. Of the post-operative traumatic osteomyelitis cases, 244 (60.5%) infections resulted from open fractures, 148 (36.7%) were post-operative infections after a closed fracture, and the mechanism of injury was unknown in 11 cases. According to the Cierny-Mader classification for 496 cases of single-site infection, sixty-seven cases (13.5%) were classified as type I, 56 cases (11.3%) as type II, 129 cases (26.0%) as type III and 244 cases (49.2%) as type IV.

### Laboratory test

Thirty-seven patients (7.4%) had a body temperature higher than 37.1 °C, 85 (16.9%) had a white blood cell count (WBC) greater than 10 000/μL, 218 (43.3%) had C-reactive protein (CRP) levels greater than 10 mg/L, and 209 (41.6%) had an erythrocyte sedimentation rate (ESR) greater than 20 mm/hr. Only 55 patients (10.9%) were positive for all three inflammation markers, and the positive rate was higher among the haematogenous osteomyelitis group than among the post-traumatic osteomyelitis group (Table 1). The positive rates for WBC and body temperature among patients without sinus infection were 26.0% and 11.3%, respectively, which were higher than for those with sinus infection (13% and 5.7%, respectively; P < 0.01 and P = 0.023), while the differences in ESR and CRP were not statistically significant.

### Pathogenic microorganism

A total of 467 patients (92.8%) had a history of antibiotic therapy before admission, and 324 (64.4%) had a positive culture; 377 strains of microorganisms were cultivated, 39 of which were cultured with two strains and 7 of which were cultured with three strains. *Staphylococcus aureus* accounted for 42.2% (159 strains), including 38 strains of MRSA. *Pseudomonas aeruginosa* accounted for 49 strains (13.0%), *Enterobacter cloacae* for 35 strains (9.2%), *E*. *coli* for 33 strains (8.8%), fungi for 7 strains (1.9%), *Acinetobacter baumannii* for 17 strains (4.5%), and other microorganisms accounted for 77 strains (20.4%). The positive rate of sinus secretion cultures was 47.9% (169/353), and 55.0% (93/169) were in accordance with the deep tissue culture. The positive rate of deep tissue cultures from post-traumatic osteomyelitis patients was 66.9% (273/408), and the proportion of *S*. *aureus* was 38.4% (117/305), while the positive rate of cultures from haematogenous osteomyelitis patients was 54.9% (50/91), and *S*. *aureus* accounted for 64.4% (38/59). Eighty-three patients had to undergo debridement more than twice before bone grafting; of these, 41 (49.4%) had positive cultures, and *S*. *aureus* accounted for 48.8% (20/41) of the microorganism distribution according to body site, as shown in Table 2.

**Table 2:** Positive rate of bacterial culture in different locations with single site patients.

Sites	Cases (Rate)	Positive rate	Rate of S. aureus	Other common bacteria
Tibia	285 (57.5%)	66% (188/285)	42.5% (80/188)	P.Aeruginosa 15.8%, Colibacillus 8.4%
Femur	133 (26.8%)	62.4% (83/133)	61.4% (51/83)	E.cloacae 8.2%, colibacillus 7.1%
Calcaneus	15 (3.0%)	73% (11/15)	45.5% (5/11)	P.Aeruginosa 27.3%, Colibacillus 9.1%
Humerus	14 (2.8%)	57% (8/14)	50% (4/8)	Colibacillus 25%, E.cloacae 12.5%
Radius and ulna	13 (2.6%)	69.2% (9/13)	44.4 (4/9)	E.cloacae 22.2%, P.Aeruginosa 11.1%
Fibula	11 (2.2%)	45.5% (5/11)	60.0% (3/5)	—
Others	25 (5.1%)	60% (15/25)	33.3% (5/15)	P.Aeruginosa 15.6%, E.cloacae 12.5%

### Treatments and consequences

All the patients had an average bone defect length of 6.8 ± 1.1 cm; 320 cases were treated with the induced membrane technique and 183 were treated with I-stage free bone grafts. No other surgical methods were used for osteomyelitis therapy. Additionally, 256 patients were fixed with internal fixation, 117 with temporary or long-term external fixation (locking compression plate, Synthes, Switzerland) and 130 patients did not undergo fixation. One hundred eight patients were treated with flaps or skin grafts.

A total of 356 patients (284 treated with the induced membrane technique and 72 treated with I-stage free bone graft) were followed for more than 18 months. The total infection control rate was 93.8% (334/356), and 94.1% (335) acquired bone union. The average union time was 6.24 ± 0.76 months. Twenty-two patients underwent repeated surgery or amputation because of recurrent infection, and 83 patients required debridement more than twice before bone grafting. The complication rate was 18.0% (64/356). There were 8 cases of nonunion, 14 cases of iliac infection, 14 cases of restricted joint activity, 7 cases of pin-track infection when external fixation was used, 5 cases of loosening fixations, and 16 cases of other complications. The infection control rate for the induced membrane technique was higher than for I-stage free bone graft. The main complication of the induced membrane technique was iliac infection, while the main complication of I-stage free bone graft was restricted joint activity. The various complications encountered in the study are tabulated in Table 3.

**Table 3:** Prognosis and complications of two different surgical methods.

Events	Induced membrane technique	I-stage free bone graft	P Value
Rate of infection control	97.2% (276/284)	80.6% (58/72)	<0.01
Average bone union time (months)	6.06 ± 0.68	7.08 ± 0.79	<0.05
Rate of debride again	18.1% (58/320)	13.7% (25/183)	>0.05
Rate of complications	18.3% (52/284)	16.7% (12/72)	>0.05
Main complication	Iliac infection (13/284)	Limited joint mobility (6/72)	—

There were no significant differences in the rate of infection control or the healing time of the bone defect according to fixation method. However, the difference in the complication rate was statistically significant (external fixation group > internal fixation group > no fixation group). The main complication in the internal fixation group was iliac infection, while pin-track infection was the main complication in the external fixation group. The prognosis and complications are elaborated in Table 4.

**Table 4:** Prognosis and complications of patients treated with induced membrane technique.

Events	Internal fixation	External fixation	No fixation	P Value
Rate of infection control	97.4% (148/152)	96.2% (100/104)	100% (28/28)	>0.05
Average bone union time (months)	6.01 ± 0.65	6.36 ± 0.70	5.28 ± 0.67	>0.05
Rate of debride again	31.0% (31/182)	22.6% (24/106)	9.4% (3/32)	>0.05
Rate of complications	16.4% (25/152)	24.0% (25/104)	7.1% (2/28)	<0.01
Main complications	Iliac infection (9/25)	Pin track infection (7/25)	Nonunion (1/2)	—

## Discussion

The incidence of osteomyelitis has changed significantly in recent decades. The aetiology and morbidity of osteomyelitis is linked to many factors, including ethnicity, lifestyle and economic conditions. In the United States, the rate of infection following fracture fixation was approximately 5%, of which 1/3 of cases transformed into refractory osteomyelitis^12^. A fracture of the tibia, age in the 50 s^13^, a BMI > 30 kg/m^2^, alcohol abuse, compartment fasciotomy, and the use of temporary external fixation are independent risk factors for bone infection. Jiang and colleagues reviewed the characteristics of osteomyelitis in southeast China^5^. China has a large population and diverse landscapes, climates and customs (lifestyles), which may contribute to the incidence and severity of disease. The current study focused on patients living in southwest China, where the humid climate and relatively poor medical facilities may contribute to higher morbidity. Our medical centre is one of few hospitals specializing in bone infection in the entire region. The study began in 2011, and it focused on the treatment and characterization of osteomyelitis. With approximately 200 million people living in the five provinces of southwest China, our data could provide regional representation of the overall situation.

The current findings suggest that post-traumatic osteomyelitis is the predominant type (80.1%), but haematogenous osteomyelitis is still present in large proportions, particularly with a childhood and adolescent onset^14^. The average age of the haematogenous osteomyelitis patients was 35.4 years due to chronic osteomyelitis that was ignored for a long time, while normal immunized adults (38.9%) were seen at first onset. The most common site of osteomyelitis infection was the tibia, and 28.6% of cultures from the tibia were positive^10^, while the most common site of haematogenous osteomyelitis was the femur, and the positive rate of microbial cultures from the femur was 54.9%. These findings show that the characteristics of osteomyelitis vary significantly in different regions and according to work and/or race.

The recommended standard for osteomyelitis diagnosis consists of biopsy and deep tissue culture^15^. Damaged bones and soft tissues may expose numerous proteins, such as collagen and fibronectin, which serve as a substrate for bacterial attachment and growth^1^. A bacterial culture shows positive growth and may be affected by culture time, culture conditions, antibiotics use before culture, and biofilm-associated bacterial strains^16^. In the present study, the positive rate of the cultures was 64.4% after a normal duration (5 days), which differs slightly from previous reports^5^. The conventional culture time is approximately 1 week but in case of partial cultures may take approximately 2 weeks^5^. In contrast, the types and phenotypes of the microorganism may change as the disease progresses. A very large number of patients (92.8%) had undergone heavy antibiotic treatment before admission to the hospital, which may have led to changes in drug resistance. For patients with sinus infection, the level of sinus infection secretion culture was limited; the positive culture rate was lower (47.9%) than the overall positive rate (64.4%) in the current study. The percentage of microbial species shared between sinus infection secretion cultures and deep tissue culture was only 44.0%.

*Staphylococcus aureus* is abundant in the skin microflora and in mucus and is a frequent cause of biofilm-associated infections^17,18^. A study by Arias^4^ examined 193 patients with *S*. *aureus* (28.7%), MRSA (9.3%), and polymicrobial strains (31%), but the current study found higher rates of *S*. *aureus* (37.3%), including an 11.7% MRSA rate and a14.2% rate of polymicrobial infection. Among the patients with monomicrobial infection, the proportion of *S*. *aureus* was considerably higher (76.2%) in the haematogenous osteomyelitis patients than in the post-traumatic osteomyelitis patients (43.6%), and the rates of *Escherichia coli* and *Enterobacter cloacae* colonies were gradually increased.

For typical osteomyelitis, diagnosis following a patient history and physical examination is not difficult. However, for some atypical cases, preoperative auxiliary examinations are needed to obtain a preliminary diagnosis, and the value of serum inflammation markers is limited by many factors (cold, pain, surgery and other factors). Positive rates of WBC (16.9%), CRP (43.3%) and ESR (41.6%) were found for only a small number of patients (10.9%), indicating that positive inflammatory markers have poor specificity. The trending levels of acute inflammatory markers is more significant and indicates the infection status following initiation of treatment^19^. The levels of inflammatory markers gradually ascend 2 weeks post operation, indicating the failure of infection control.

To treat osteomyelitis, surgical intervention, conservative treatments or a combination of both can be used depending on the physician’s viewpoint^4^. The results of the present study show that the average duration of post-traumatic osteomyelitis was 37.1 ± 9.05 months and the average number of operations was 2.6 ± 0.27, while for haematogenous osteomyelitis, the average duration was 174.9 ± 16.14 months and the average number of operations was 1.1 ± 0.20. This indicates that physicians prioritize surgical approaches for post-traumatic osteomyelitis, while conservative treatment is more often adopted for haematogenous osteomyelitis.

The prognosis of osteomyelitis is related to its classification and treatment and the patient’s socioeconomic status^20^. Conventional techniques, such as vascularized fibula autograft and Iliazarov bone transport, yield poor long-term outcomes, often due to graft resorption and revascularization by creeping substitution^21,22^. For patients with bone defects greater than 4-5 cm, bone grafting treatment may lead to absorption of the grafts. May *et al*.^23^ reported that for tibial infections requiring bone graft for structural support, the repair time for the bone defect was 3–6 months, while tibial defects > 6 cm and intact fibula reconstruction require 12–18 months. The average bone defect length for our patients was 6.8 ± 1.1 cm, and the average bone healing time was 6.24 ± 0.76 months. The speed of the bone defect repair and the infection control rate were superior with the induced membrane technique compared with I-stage free bone graft, and the incidence of complications was not significant.

The use of the induced membrane technique to repair bone defects has been widely adapted in recent years because of its simple operation and high healing rate^24–26^. The complications associated with this technique include infection recurrence, delayed stress fracture and nonunion of bones^27,28^. Our results indicated that the most common complication was iliac infection, which may relate to the improper handling of the bone supply area. There were no significant differences in the rate of infection control and the bone defect healing time according to fixation method. However, there was a significant difference in the complication rate, suggesting that the use of internal fixation may not necessarily increase the incidence of infection. In contrast, external fixation may increase the incidence of complications, particularly pin-track infection; therefore, we propose the use of internal fixation, particularly for infection control.

This study had certain limitations; for example, diabetic foot infection cases were not treated at our department, and we did not have complete follow-up data, which impacted the observation of the overall distribution of osteomyelitis. We applied logistic regression analysis with the aim of identifying risk factors (gender, age, smoking, sinus and other factors) for osteomyelitis and infection control, but the results did not permit any clear conclusions. We did not find any connection between risk factors and a reduced recurrence of infection.

## Conclusion

In southwest China, post-traumatic osteomyelitis is the most common type of osteomyelitis, but the incidence of haematogenous osteomyelitis is still higher than developed regions in the country. *S*. *aureus* is the main bacterial strain for bone infections. The speed of bone defect repair and the infection control rate of the induced membrane technique was superior to that of I-stage free bone graft. Internal fixation should be given priority as it does not increase the rate of infection recurrence but reduces the risk of complications such as pin-track infection.
